# Familial inheritance of the 3q29 microdeletion syndrome: case report and review

**DOI:** 10.1186/s12920-019-0497-4

**Published:** 2019-03-18

**Authors:** Wahab A. Khan, Ninette Cohen, Stuart A. Scott, Elaine M. Pereira

**Affiliations:** 1 Department of Genetics and Genomic Sciences, Icahn School of Medicine at Mount Sinai, New York, NY 10029 USA; 2 Sema4, a Mount Sinai venture, Stamford, CT 06902 USA; 3 Division of Cytogenetics and Molecular Pathology, Donald and Barbara Zucker School of Medicine at Hofstra Northwell, Northwell Health Laboratories, Lake Success, New York City, NY 11020 USA; 4 Department of Pediatrics, Division of Clinical Genetics, New York-Presbyterian Morgan Stanley Children’s Hospital, Columbia University Medical Center, New York, NY 10032 USA

**Keywords:** 3q29 microdeletion, Developmental disabilities, Neuropsychiatric phenotypes familial inheritance, Copy number variation

## Abstract

**Background:**

The chromosome 3q29 microdeletion syndrome is characterized by a clinical phenotype that includes behavioral features consistent with autism and attention deficit hyperactivity disorder, mild to moderate developmental delay, language-based learning disabilities, and/or dysmorphic features. In addition, recent data suggest that adults with chromosome 3q29 microdeletions have a significantly increased risk for psychosis and neuropsychiatric phenotypes.

**Case presentation:**

We report a 3-year-old male with global developmental delay, anemia, and mild dysmorphic facial features. Clinical chromosomal microarray (CMA) testing of the proband detected a heterozygous 1.21 Mb deletion at chromosome 3q29, consistent with a diagnosis of the 3q29 microdeletion syndrome. Interestingly, subsequent parental testing determined that the pathogenic deletion was inherited from his otherwise healthy mother who had a history of learning disabilities. The chromosome 3q29 microdeletion was not detected in the healthy older sibling of the proband by CMA testing, nor was it prenatally detected in a subsequent maternal pregnancy.

**Conclusion:**

Our report highlights the 3q29 microdeletion syndrome as an illustrative example of the importance of a molecular diagnosis for families that harbor pathogenic copy number aberrations with variable expressivity, in particular those that also impart an increased risk for adult onset neuropsychiatric phenotypes.

## Background

Chromosomal microarray (CMA) testing offers a powerful approach to detect submicroscopic copy number changes in the human genome [1, 2], which has led to the discovery of several microdeletion and microduplication syndromes [3, 4]. Moreover, clinically-relevant intragenic copy number changes are now also being discovered by large-scale genome sequencing efforts [5–7]. Importantly, some of these syndromes have incomplete penetrance and/or variable expressivity, which often result in nonspecific neurodevelopmental clinical features and challenging clinical diagnoses. The chromosome 3q29 microdeletion syndrome is characterized by a clinical phenotype that can include behavioral features consistent with autism and attention deficit hyperactivity disorder, mild to moderate developmental delay, and language-based learning disabilities [8]. A subset of cases also present with microcephaly and mild dysmorphic features (e.g., elongated face, long fingers, and joint laxity) [9, 10].

The majority of 3q29 microdeletion syndrome cases occur de novo [11–15], which are most likely derived by the regional low copy repeats that act as substrates for non-allelic homologous recombination [8]. The recurrent 3q29 microdeletion spans ~ 1.6 Mb with molecular boundaries encompassing the *PAK2* and *DLG1* candidate genes [8, 16]. Of note, inheritance from a mildly affected parent has also been reported among several independent families, including this index case (Table 1) [11, 14, 17–20]. This variable expression suggests that the incidence of the 3q29 microdeletion syndrome may potentially be higher than previously estimated (1 in 30,000-40,000) [21], as the constellation of features among certain families may not be clinically overt. Importantly, the 3q29 microdeletion is also one of the largest known risk factors for schizophrenia (~ 40 fold), surpassing even the recurrent 22q11.2 microdeletion syndrome [16]. As such, accurate detection and molecular diagnosis of the 3q29 microdeletion syndrome is critical for proper genetic counseling and when providing neuropsychiatric risk information to affected family members. Herein, we report a child with global developmental delay and a chromosome 3q29 microdeletion, which subsequently was found to be inherited from his otherwise healthy mother who had a history of learning disabilities. This informative case underscores the utility of CMA testing for non-specific neurodevelopmental pediatric indications and highlights the 3q29 microdeletion syndrome as an illustrative example of the importance of a molecular diagnosis for pathogenic copy number aberrations with variable expressivity.

**Table 1:** Clinical features of inherited 3q29 microdeletion syndrome cases identified through affected probands

	Current study		Monfort et al. 2008 [18]		Ballif et al. 2008 [17]		Li et al. 2009 [14]		Digilio et al. 2009 [19]		Digilio et al. 2009 [19]		Clayton-Smith et al. 2010 [11]		Petrin et al. 2011 [20]	
Inheritance	Maternal		Maternal		Maternal		Paternal		Maternal		Maternal		Maternal		Paternal**	
GRCh37/hg19 Position (Mb)	195.8–197.0		not provided		recurrent deletion		195.9–197.3		195.7–197.3		195.7–197.3		recurrent deletion		195.7–197.3	
Clinical features	P1	PAR	P2	PAR	P3/4¥	PAR	P5	PAR	P6	PAR	P7	PAR	P8/9*	PAR	P10	PAR
Autistic features	–	–	–	–	+	–	–	–	–	–	–	–	+	–	–	–
Broad or high nasal root	+	+	+	–	+	–	–	–	–	–	–	–	+	+	+	–
Cardiac anomaly	–	–	–	+	–	–	+	+	–	–	+	–	–	–	–	–
Chest wall deformity	–	–	–	–	+	–	–	–	–	–	–	–	+	–	–	–
Cleft lip w/ or w/o cleft palate	–	–	–	–	–	–	–	–	–	–	–	–	–	–	+	–
Cognitive deficit	+	–	+	+(mild)	+	–	–	–	+	–	+(mild)	–	+	+	–	–
Developmental Delay	+	–	+	–	–	–	+	+(mild)	–	–	+	+	+	+	+	–
Delayed language/learning	+	+	+	–	+	–	+	–	+	+(mild)	+	+	+	+	–	–
Palpebral fissure defects	+	–	–	–	–	–	+	–	+	+	+	–	–	–	–	–
Ear anomalies	+	+	+	–	+	–	+	–	–	+	–	–	–	–	+	–
Feeding difficulties	–	–	–	–	–	–	–	–	–	–	+	–	–	–	–	–
GI problems	–	–	–	–	–	–	+	–	–	–	–	–	–	–	–	–
Microcephaly	–	–	–	–	+	–	–	–	+	+	+	+	+	+	–	–

+, present; −, not mentioned/evaluated; *P*, patient, *PAR* parent

¥ patients 3/4 specific clinical features were not provided; clinical features tabulated here for patients 3/4 were reported as common findings in Ballif et al. 3q29 microdeletion cohort

* patients 8/9 are siblings (maternal grandmother of P8/9, not shown in table, had the same 1.6 Mb microdeletion – noted as the 11th case of parental transmission in a multi-generation family)

**reported as a mosaic loss in nonaffected father of proband

Coordinates of the recurrent deletion are approximately chr3:195,756,054-197,344,662 (GRCh37/hg19; ISCA region-37,443)

## Case presentation

**Fig. 1:**
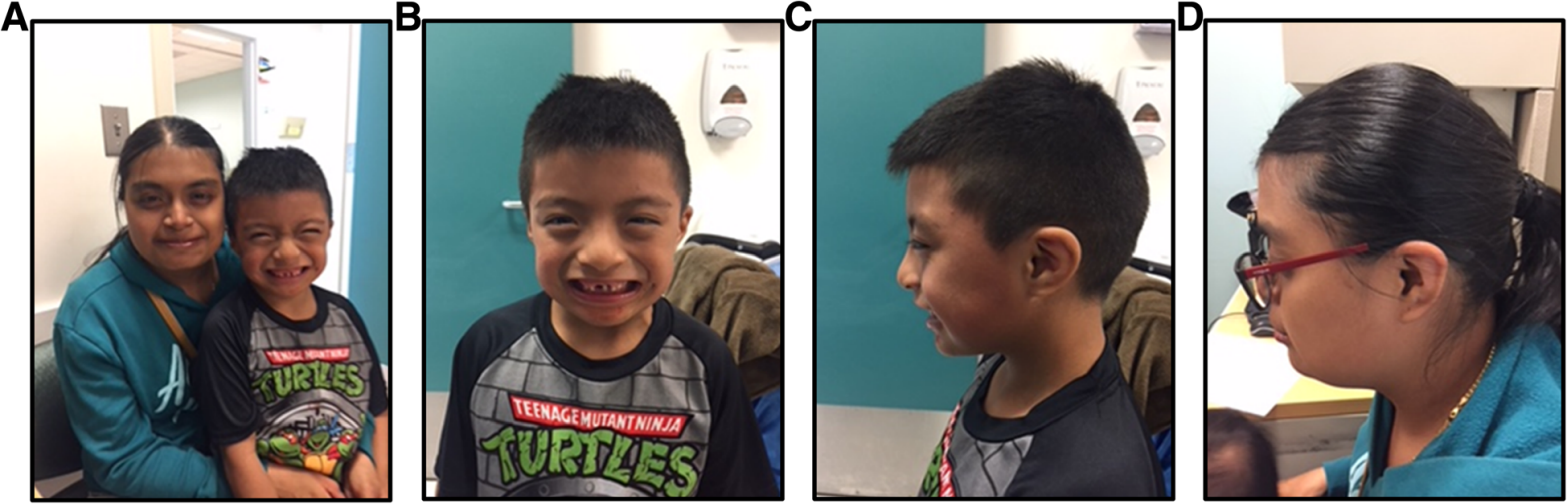
Facial features of the proband and his affected mother. Note the similar broad nasal bridge seen in both the parent and proband (**a**). A closer look at the proband reveals periorbital puffiness and slightly downward slanting palpebral fissures (**b**). Profile views of the proband and his mother demonstrate similar ears with an overfolded helix (**c**-**d**)

The 3-year-old male proband was referred to Clinical Genetics for evaluation of a history of developmental delay. He was the second child of non-consanguineous parents of Mexican descent, and his pedigree was remarkable for a mother and maternal male first cousin who required special education mainly for learning disabilities. The prenatal history included a maternal chlamydia infection at 3 months gestational age (treated with antibiotics) and intrauterine growth restriction. He was born vaginally at 37 weeks gestational age, weighing 1.98 kg, and spent a total of 23 days in the NICU requiring nasogastric feeds and phototherapy. He walked and began to speak at 14 months of age and was subsequently diagnosed with global developmental delay by a behavioral pediatrician. He began early intervention services at the age of 2 years, receiving occupational, physical and speech therapy. At 3 years of age he was placed in special education classes getting speech and occupational therapies. At the time of his initial genetics evaluation, the patient could understand directions and point to body parts. He did not know the alphabet, colors, or numbers. In addition to these developmental delays, the proband has a history of anemia that is followed by his pediatrician. On physical exam, the patient was found to have a low anterior hair line. His palpebral fissures were slightly downward slanting, and infraorbital puffiness was noted. He has overfolded helices bilaterally, a broad nasal bridge, a wide grin and thin upper lip, which generally resembled the facial features of his mother (Fig. 1).

**Fig. 2:**
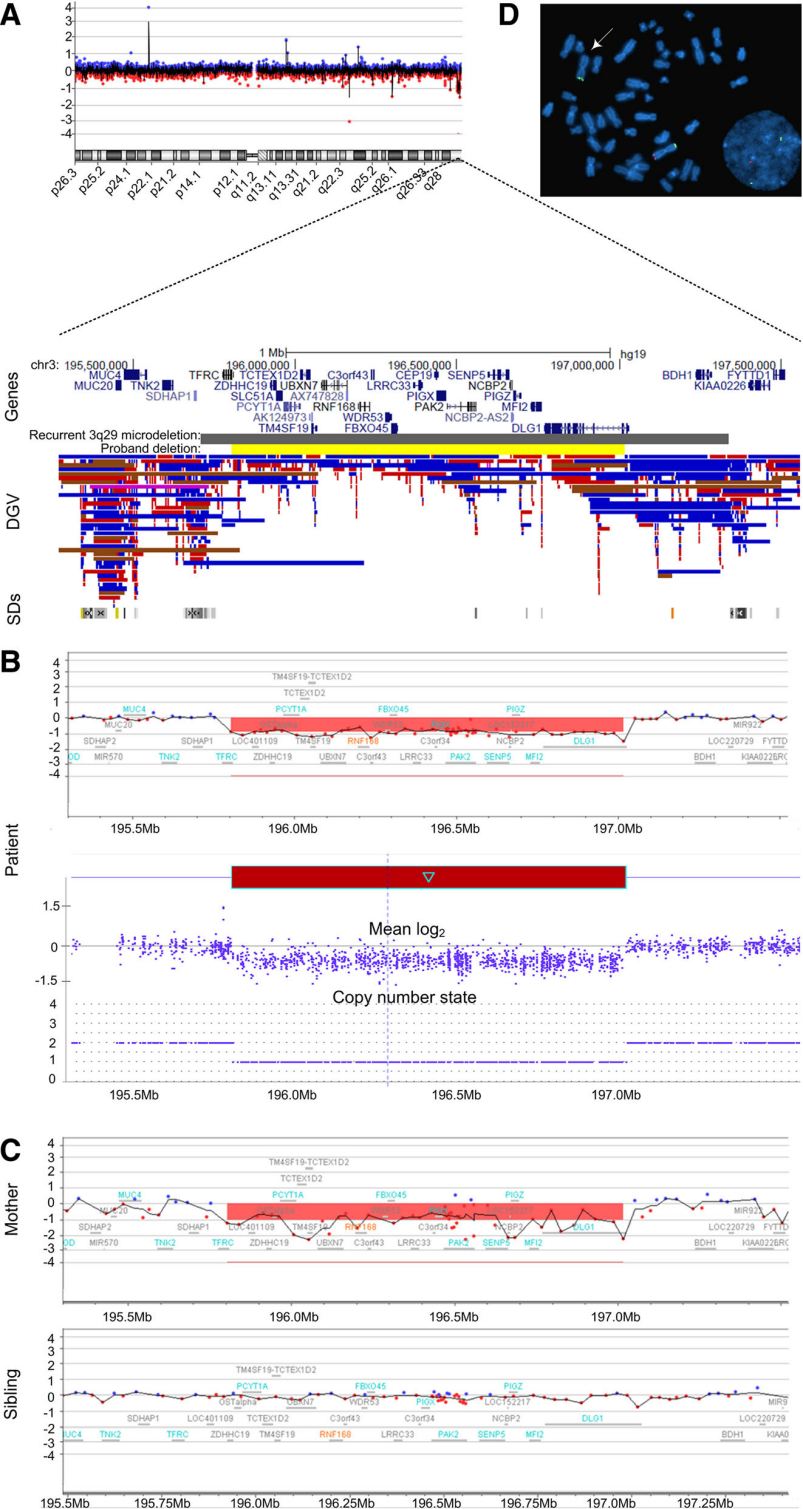
Chromosome view showing the subtelomeric 3q29 microdeletion region. Each red and blue dot represents an oligonucleotide probe along the length of chromosome 3 with its cytobands shown on the x-axis and the log_2_ ratio plotted on the y-axis. The dotted lines provide a focused view of a portion the 3q29 cytoband, annotations of UCSC genes (hg19) (http://genome.ucsc.edu/), user-defined tracks indicating the recurrent 1.6 Mb deletion interval [(reported in [10]], and the 1.21 Mb deletion reported in our index patient. Immediately below, the presence of copy number variants (CNVs) annotated in the Database of Genomic Variants (DGV; blue: gain; red: loss) and region-specific segmental duplications (SDs) are shown in context (**a**). Zoomed-in views of chromosomal microarray (CMA) plots (X axis: hg19 genomic coordinates; Y axis: mean log_2_ ratio; Agilent Technologies) depicting heterozygous copy number loss in the proband (top panel). Refinement of the deletion with a higher resolution CMA platform (Affymetrix; bottom panel arr[GRCh37]3q29(195806608_197029439)× 1) (**b**). CMA plots of the mother detailing the same heterozygous deletion as in the proband, whereas the sibling did not inherit the pathogenic chromosome 3q29 deletion (**c**). Metaphase and interphase FISH specific to the subtelomeric region of chromosome 3q (D3S4560 Abbott Molecular TelVysion, red signal), and an internal control probe specific to the subtelomeric region of chromosome 3p (D3S4559 Abbott Molecular TelVysion, green signal) in the proband. A red signal is absent at 3q29 on one of the chromosome 3 homologs (arrow), which confirms the heterozygous deletion identified by CMA testing (**d**)

Clinical Fragile X testing on DNA isolated from the proband was normal with 29 CGG repeats; however, CMA testing using the SurePrint G3 ISCA CGH + SNP 4 × 180 microarray (Agilent Technologies, Santa Clara, CA) [22, 23] detected a heterozygous 1.21 Mb deletion of chromosome 3q29 (reported as arr[GRCh37] 3q29(195804728_197016624)× 1) (Fig. 2). This microdeletion is smaller than the 1.6 Mb recurrent 3q29 microdeletion defined above, but still nested within its segmental duplication boundaries (Fig. 2). It included 28 genes and transcripts, and had only minimal overlap with copy number variants (CNVs) reported among healthy individuals in the Database of Genomic Variants (DGV; http://dgv.tcag.ca) [24]. The interstitial chromosome 3q29 microdeletion was confirmed using the higher resolution CytoScan® HD platform (Affymetrix, Santa Clara, CA) and metaphase fluorescent in situ hybridization (FISH) using subtelomeric DNA probes (Fig. 2). Notably, familial CMA testing determined that the pathogenic chromosome 3q29 microdeletion was maternally inherited and not present in the healthy older sibling (Fig. 2), nor was it prenatally detected in a subsequent maternal pregnancy.

## Discussion and conclusions

The significance of structural variation in human disease and phenotypic diversity has increasingly become recognized, and several genomic studies have generated catalogs of CNVs to facilitate a better understanding of their clinical relevance [3, 25, 26]. It is estimated that up to 60% of the human genome may contain structural variants in the form of CNVs in the general population, which typically range in size from 100 bp to 50 kb [27], and clinical interpretation of these aberrations when identified by CMA testing is enabled by medical genetics practice guidelines [28]. Larger copy number aberrations are more likely to be pathogenic, as are de novo variants; however, large-scale genomic studies on clinically relevant sequence variants and CNVs are increasingly underscoring the importance and interpretation challenges of incomplete penetrance and variable expressivity [3, 29–31]. We report a case of the 3q29 microdeletion syndrome in a 3-year-old male with developmental delay, which was found to be inherited from an otherwise healthy parent with a history of learning disabilities (Table 1). This educational case is an illustrative example of the importance of a molecular diagnosis for children with non-specific neurodevelopmental indications, particularly for families that harbor pathogenic copy number aberrations with variable expressivity.

As indicated above, the recurrent 3q29 microdeletion syndrome has a heterogeneous clinical phenotype, and is enriched among young adults with psychiatric disorders and children with developmental delay [3, 32]. In particular, mild to moderate cognitive deficits, speech delay, low birth weight, a high nasal root, and ocular abnormalities are common findings (Table 1). Other less frequent features that can present with greater variability include facial asymmetry, frontal bossing, large protuberant ears, upslanting palpebral fissures, broad nostrils, and cleft lip/palate [11–15].

The 1.21 Mb deletion identified in our proband is nested within the recurrent ~ 1.6 Mb deletion and is one of the smallest 3q29 microdeletions reported to date using a high-resolution CMA platform; however, it directly overlaps with previously described pathogenic deletions at this locus [21]. Additional 3q29 deletions nested within the recurrent microdeletion have also been described, either with a BAC or low resolution oligonucleotide microarray platform [12, 13, 17, 33]. A common pattern of moderate learning problems, speech delay, psychiatric disorder, and autistic features constitutes the primary phenotypic overlap in this cohort of smaller 3q29 deletions. A comparison of the genomic intervals between the 1.21 Mb deletion in our proband, the previously reported smaller deletions [12, 13, 17, 33], and the more common recurrent 1.6 Mb deletions indicates that all include the *PAK2* candidate gene. The centromeric region of all deletions share the *TFRC* gene, which is also disrupted by the deletion in our proband and focally deleted in the recurrent deletion (Fig. 2). The telomeric region commonly includes *DLG1*; however, the recurrent larger deletions extend past *DLG1* and into *BHD1* (Fig. 2). Haploinsufficiency of these genes, in particular *DLG1*, has been implicated in some forms of schizophrenia and the behavioral characteristics of the 3q29 microdeletion syndrome [16, 17, 34].

In conclusion, our case presents a set of phenotypic features that add to the heterogeneous expression of the 3q29 microdeletion syndrome. In particular, the finding of anemia in our proband is uncommon among reported 3q29 microdeletion syndrome cohorts; however, given that the co-occurrence of anemia may be coincidental, additional patients are needed to confirm whether this is a rare feature of the 3q29 microdeletion. The familial inheritance of this pathogenic deletion and the phenotype of the transmitting parent is summarized with other reported familial 3q29 microdeletion syndrome cases in Table 1. Consistent among these families is a milder phenotype in the transmitting parent, often restricted to mild dysmorphic features and/or a history of learning/language delays. As the landscape of genomic variation in different populations continues to be defined, the importance of incomplete penetrance and variable expressivity are increasingly emerging. As such, the familial inheritance of this pathogenic deletion highlights both the clinical relevance of variable expressivity in genetic disease and the importance of clinician awareness, particularly for inherited aberrations that also impart an increased risk for adult onset neuropsychiatric phenotypes.

## Abbreviations

CMA: chromosomal microarray
CNV: copy number variant
DGV: Database of Genomic Variants
FISH: fluorescence in situ hybridization
